# Combined effect of cigarette smoking and non-ferrous metal exposure in the development of cardiovascular disease in industry workers: a case-control study

**DOI:** 10.1186/1617-9625-12-S1-A31

**Published:** 2014-06-06

**Authors:** Eniko Viragh, Karoly Viragh, Claudia Munteanu

**Affiliations:** 1 University of Medicine and Pharmacy, Targu Mures, 540139, Romania; 2 Olive View UCLA Medical Center, Sylmar, Los Angeles, California, 91342, USA; 3 Authority of Public Health, Sibiu, 550012, Romania

## Background

The purpose of the study was (1) to determine the prevalence of smoking and cardiovascular disease in workers in non-ferrous metallurgy, and (2) to evaluate the effect of cigarette smoking in the development of cardiovascular disease in exposed workers.

## Materials and methods

A retrospective case-control study was performed. Industry workers from a nonferrous plant and controls were monitored for an 8-year period. During this period, all workers received regular clinical examinations, which included evaluation for smoking status, occupational exposure to noxious non-ferrous metals (lead,Pb; cadmium,Cd), and cardiovascular disease (hypertension, coronary artery disease, peripheral vascular disease) using an epidemiological questionnaire . Four representative groups were selected: Group (1) included all workers with both smoking and exposure to noxious non-ferrous metals. Group (2) included workers with smoking but without noxious metal exposure. Group (3) included workers without smoking but with exposure to noxious metals. Group (4) included workers without smoking and without exposure to noxious metals. Groups (2) and (4) were selected in such a way to match Groups (1) and (3) by age, gender, work history, and lifestyle. The prevalence of smoking and cardiovascular disease was determined in each group. Linear regression analysis was used to assess the correlation between the levels of exposure and biomarkers of exposures, as well as between the amount of smoking and the burden of cardiovascular disease.

## Results

During the studied period, noxious non-ferrous metal (Pb and Cd) levels in the air of all workplaces were persistently high (Pb=0.9-13.3mg/m3; Cd=0.3-1.3mg/m3). Clinical examination identified the classic symptoms of chronic occupational intoxication with Pb and Cd. There was a relatively high prevalence of smoking in each group. The prevalence of cardiovascular disease was significantly higher in smokers and exposed workers. Linear regression analysis identified a strong positive relationship between the levels of exposure and biomarkers of exposure(r=0.69), between the amount of smoking and burden of cardiovascular disorders (r=0.65).

## Conclusions

There is high prevalence of smoking and cardiovascular disease in industry workers. Cigarette smoking is an important risk factor for cardiovascular disease and acts in combination with noxious non-ferrous metals in exposed industry workers. Cigarette smoking may act as a confounder in the assessment of the severity of occupational disease related to noxious metal exposure in industry workers. The goal for all facilities and workers is to minimize smoking and occupational exposure to noxious agents.

